# Correction: From Innovation to Diversification: A Simple Competitive Model

**DOI:** 10.1371/journal.pone.0144564

**Published:** 2015-12-03

**Authors:** The PLOS ONE Staff

There are errors in the Funding section. The correct funding information is as follows: Fabio Saracco has been paid by Crisis Lab in the first submission, and Riccardo Di Clemente has been paid by Growthcom Project FP7, Grant Agreement 611272.

The publisher apologizes for the error.
