# Supplementary material for: From Innovation to Diversification: A Simple Competitive Model
Source: PLoS One. 2015 Nov 6;10(11):e0140420. doi: 10.1371/journal.pone.0140420 (PMC4636243; doi:10.1371/journal.pone.0140420)
Supplement: S1 File — The supporting information with the mathematical appendix, and the description on the tuning parameter methodology. (PDF) [file pone.0140420.s001.pdf]

Supporting Information

From innovation to diversification: a simple competitive model

Fabio Saracco[1], Riccardo Di Clemente[1] *, Andrea Gabrielli[1,2] & Luciano Pietronero[1,3,4]

[1] *Istituto dei Sistemi Complessi - CNR, Via dei Taurini 19, 00185 Rome, Italy*

[2] *IMT Institute for Advanced Studies Lucca, Piazza S. Ponziano 6, 55100, Lucca, Italy*

[3] *Sapienza, Università di Roma, Piazzale A. Moro 5 Roma Italy.*

[4] *London Institute for Mathematical Sciences, 35a South St, Mayfair London United Kingdom.*

*Corresponding author: *E-mail address*: riccardo.diclemente@imtlucca.it

Appendix

Fitness and Complexity

In order to extract the information contained in the $\hat{M}$-matrix, the authors of [1, 2, 3, 4], overcoming flaws present in the seminal works [5, 6], propose a metric for countries and products, the celebrated Fitness and Complexity algorithm: this recursive and non linear algorithm is a sort of PageRank applied to bipartite networks, where Fitness is the quantity for countries, while Complexity is the one for products. The idea at the the basis of the algorithm is that highest fitness countries are those which are able to export the highest number of the most exclusive products, i.e. those with the highest complexity. In particular, the Fitness F_c for the generic country c and Quality Q_p for the generic product p at the $n-$th step of iteration, are defined as

$$\begin{cases} \tilde{F}_c^{(n)} = \sum_p m_{cp} Q_p^{(n-1)} \\ \tilde{Q}_p^{(n)} = \dfrac{1}{\sum_c m_{cp} \frac{1}{F_c^{(n-1)}}} \end{cases} \rightarrow \begin{cases} F_c^{(n)} = \dfrac{\tilde{F}_c^{(n)}}{\langle \tilde{F}_c^{(n)} \rangle} \\ Q_p^{(n)} = \dfrac{\tilde{Q}_p^{(n)}}{\langle \tilde{Q}_p^{(n)} \rangle} \end{cases}, \tag{S1}$$

where the symbols $\langle \cdot \rangle$ indicate the average taken over the proper set. The initial condition are taken as $F_c^0 = Q_p^0 = 1$ $\forall c \in N_c$, $\forall p \in N_p$, where N_c and N_p are the number respectively of countries and products (the convergence of the algorithm described by Eqs.(1) depends on the shape of the matrix $\hat{M}$, as it has been discussed in [7]).

Non trivial benchmarks

Fitness and Quality distributions

Using Fitness and Complexity it is possible to reveal several non-trivial properties of the $\hat{M}$-matrix: the very first observation is that, once reordered rows and columns respectively by fitness and complexity, the $\hat{M}$-matrix shows a peculiar triangular form, already observed in biological systems, [8, 9, 10, 11] . The triangular shape of $\hat{M}$ shows that even the most diversified countries do not export just the most exclusive products, but even the common ones [1, 2, 3, 4, 5, 6, 7, 12, 13].
The form of the distribution for the Fitness (Quality) ranking against country diversification (products ubiquity) depends strongly on the shape of $\hat{M}$. They are sparse distributions, due to the non-linearity of the algorithm of Fitness/Complexity, which cause even the peculiar shape, as shown in the Fig. 3(a, b) (main paper): real data are blue points, while the cloud represent the frequency of the simulated data.

Nestedness

As already mentioned, the "triangularity" of the $\hat{M}$-matrix is a typical feature of biological mutualistic networks. Traditionally, in the biology literature the triangular form of the biadjacency matrix is measured by the *nestedness*, i.e. how much a row (or a column) is a subset of the others. In the literature there is a great amount of different proposals for the nestedness definition, [8, 9, 11, 10, 14]; here we decide to use the NODF (*Nestedness metric based on Overlap and*

Decreasing Fill) presented in [8] because, according to us, it is the most intuitive. Using the definition of Eqs. 3,

$$T^r_{cc'} \stackrel{\text{def}}{=} \begin{cases} k_c \neq k_{c'} & \dfrac{\sum_p m_{cp}\, m_{c'p}}{\text{Min}\{k_c, k_{c'}\}} \\ \\ \text{otherwise} & 0 \end{cases} ; \tag{S2}$$

$$T^c_{pp'} \stackrel{\text{def}}{=} \begin{cases} k_p \neq k_{p'} & \dfrac{\sum_c m_{cp}\, m_{cp'}}{\text{Min}\{k_p, k_{p'}\}} \\ \\ \text{otherwise} & 0 \end{cases} . \tag{S3}$$

The total nestedness measure NODF_t is then

$$\text{NODF}_t \stackrel{\text{def}}{=} 2\frac{\sum_{c<c'}^{N_c} T^r_{c\,c'} + \sum_{p<p'}^{N_p} T^c_{p\,p'}}{N_c(N_c-1) + N_p(N_p-1)}, \tag{S4}$$

where N_c and N_p are the number respectively of countries and products. Note that the final value of the nestedness gets contribution just in case the number of the non-zero elements of the two rows (or columns) considered is different (for that, the name "decreasing fill" in the NODF acronym). Eventually, the final formula for the nestedness is the weighted sum of the contributions from rows and from columns, so it is possible to isolate the two different nestedness for rows (i.e. countries, NODF_c) and for columns (i.e. products, NODF_p):

$$\text{NODF}_c \stackrel{\text{def}}{=} 2\frac{\sum_{c<c'} T^r_{c\,c'}}{N_c(N_c-1)}; \qquad \text{NODF}_p \stackrel{\text{def}}{=} 2\frac{\sum_{p<p'} T^c_{p\,p'}}{N_p(N_p-1)}. \tag{S5}$$

Since usual matrices $\hat{M}$ are quite "rectangular", i.e. the number of products is much greater then the number of countries, the total NODF 4 is biased by the contribution from the products. In fact, combining Eqs.4 and 5:

$$\text{NODF} = \frac{N_c(N_c-1)\text{NODF}_c + N_p(N_p-1)\text{NODF}_p}{N_c(N_c-1) + N_p(N_p-1)} \stackrel{N_p \gg N_c}{\simeq} \text{NODF}_p. \tag{S6}$$

Because of the previous relation, for the analysis of the initial conditions we just compared the results for NODF_c and NODF_p, while the effect of the previous approximation is shown in the comparison between the Table S1 and the Table S3.

Assortativity

The assortativity parameter r [15] has been introduced in order to measure how much nodes tend to be linked by nodes with a similar degree. More in details, r takes values from -1 to 1, where -1 denotes a network perfectly disassortative, i.e. lowest degree nodes are linked to the highest degree ones, while 1 denotes a network perfectly assortative, i.e. high degree vertices links with highest degree one; in terms of the adjacency matrix and node degrees, r can be written as

$$r \stackrel{\text{def}}{=} \frac{2M\big(2\sum_{c,p} k_c m_{cp} k_p - (\sum_c k_c^2 + \sum_p k_p^2)^2\big)}{2M\big((\sum_c k_c^3 + \sum_p k_p^3) - (\sum_c k_c^2 + \sum_p k_p^2)^2\big)}. \tag{S7}$$

For the previous discussions about the triangularity, one may expect that the value of assortativity is negative (since poorly diversified countries exports the most ubiquitous products), but with a low absolute value, say much less than 0.5, since high degree countries have low complexity products in their export basket, as well as more complex ones. This expectation is just partly satisfied, since the value of r for the matrices observed is indeed negative, but its absolute value is quite large, say of the order of 0.6: at a second look at the Fig. 3(c) (main paper), in effect, it is possible to see that the density of the export basket of most diversified countries moves toward the low degrees products, i.e. the most exclusive ones.

Motifs for bipartite networks

Some motifs for bipartite networks have been defined in the contest of biological mutualistic networks. One of the most used is the checkerboards score [16] , i.e. the number of patterns of 2×2 submatrices present in the biadjacency matrices as mutually-exclusive terms such as ▚, ▞[1,2]. The checkerboards score, in other words, measures the how much mutually exclusive are the choices made by different countries about the composition of their export basket. The total number of possible checkerboards patterns can be written as

$$N_{\text{checkerboards}} \stackrel{\text{def}}{=} \frac{1}{2} \sum_{c,c'} \sum_{p,p'} m_{cp}(1 - m_{cp'})(1 - m_{c'p})m_{c'p'}. \tag{S8}$$

Other several motifs for bipartite networks have been proposed in [17, 18] in order to uncover the structural properties of the system at hand. Among others, we decide to focus on V and Λ motifs[3]. Respectively, the total number of $V-$motifs and $\Lambda-$motifs count the total co-occurrence of products in two different export baskets and of countries in the set of producer of two different goods; in term of the entries of the biadjacency matrix $\hat{M}$, they are defined as

$$\begin{aligned} N_V &\stackrel{\text{def}}{=} \sum_{c<c'} \sum_{p} m_{cp} m_{c'p}; \\ N_\Lambda &\stackrel{\text{def}}{=} \sum_{p<p'} \sum_{c} m_{cp} m_{cp'}. \end{aligned} \tag{S9}$$

Tuning parameters

The model, described in details in section "The model", has a four-dimensional parameter space. In order to determine which of the values of $(\alpha,\ \beta,\ \gamma,\ k^0)$ are more compatible with observations we generated matrices for all possible values of the parameters and compared the results with the observed data. In particular, we focus on initial conditions with $N_{\text{roots}} \leq 25$, according to the conclusions of the following section. In the Tables S1, S2, S3, S4, S5, S6, S7, simulated data have been generated for initial conditions with $N_{\text{roots}} = 20$ and $P_0 = 0.3$.

---

[1] ▚ and ▞ are meant to be 2×2 patterns in the $M-$matrix where ■ and □ represent respectively $m_{cp} = 1$ and $m_{cp} = 0$.

[2] Since we prefer absolute measures, thus not depending on the order of rows and column, in the total number of checkerboards patterns we consider the occurrence of both ▚ and ▞.

[3] The names V (Λ) come from the fact that once the two layers of the bipartite network are rotated such that the countries layer is over the products one, the former (the latter) motifs look like Vs (Λs) between the layers.

We observed that the parameter k^0 appears not to be influential on the measures for $k^0 \geq 4$. It is worth noticing that 4 is even the average value of the minimum of the ubiquity for real matrices; effectively $k_p = 4$ is the first value of the ubiquity for which the probability of a novelty exceed the probability of innovating. In the following we will present results with $k^0 = 4$; for every (α, β, γ) configuration we generated 56 matrices.

As the Tables S1, S2, S3, S4, S5 , S7 show, once β and γ are fixed, i.e. the parameters of the second and the third choices, the value of the α parameter which are able to replicate real data are quite narrow around the value $\alpha = 1.6$. Instead, for a fixed value of α, the best results are in an area around the "anti-diagonal" of the tables shown, so for decreasing β values once γ grows and vice versa.

The results for the number of $V-$motifs in Table S6 deserves a special treatment: in fact, while, the $\Lambda-$motifs are well reproduced, it happens quite rarely that the model is able to replicate the observed results. The meaning of the results reside in the definition of $V-$ and $\Lambda-$motifs: Vs (Λs) are the number of co-occurrence of countries (products) in the set of producers for every product (in the export set for every country). In effect, our algorithm is driven by a hierarchy imposed on the products, while no kind of structure is forced onto the countries set, so the constraints drive the total number of Λs, while the number of Vs is too "free".

Tuning Initial Conditions

In Table S8 we compare different values of N_{roots} from 10 to 40 (at steps of 5 roots) and different P_0, from 0.2 to 0.6 (at steps of 0.05); we fix the values of the parameter for the evolution dynamics among the best performing ones, according to the previous analyses, i.e. $\alpha = 1.6$, $\beta = 1$, $\gamma = 0.6$ and $k^0 = 4$; for every initial conditions configuration we produced 50 simulated $M-$matrix. The results can be observed in the Table S8: for low values of N_{roots}, the discrepancy among different distributions relative to different values of P_0 is limited and very often cross the red line representing real data. Because of it, we mostly used low $N_{\text{roots}} \leq 25$, since they need less fine tuning on the P_0. Moreover, higher P_0 are less precise, especially for a higher number of roots: thus it seems that the mean number of roots per countries to make the algorithm start should be quite small, say ~ 6.

We tried, imposing some offsets for every choice, even to make the algorithm start from no product, but the result are not satisfying since the usual measures on the matrix are not replicated.

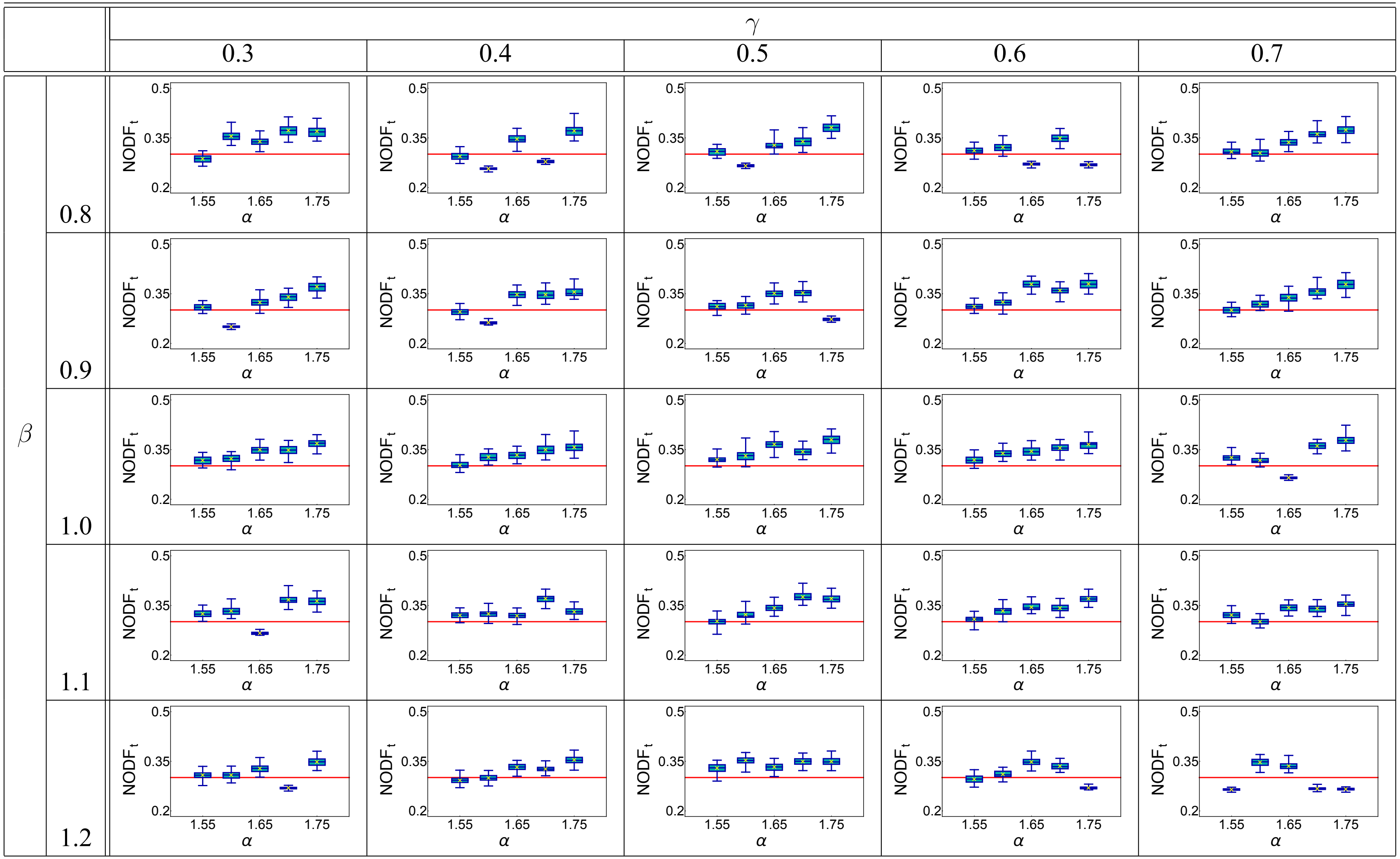

Table S1: Parameters space analysis: $\mathbf{NODF_t}$. It is possible to observe the variation of the NODF_t at the changing of the parameters α, β γ; the parameter k^0 has been kept fixed to the value 4, since no variation in any of the measure analysed has been observed for greater values. The NODF_t measured on the original matrix is represented as a red line. The best values of the parameter α are the lowest analysed, i.e. $\alpha \leq 1.65$. Instead, the wider area of acceptance for the γ parameters is for the central area of the table, i.e. $0.4 \leq \gamma \leq 0.6$, while β is more or less non-influential on the the acceptance of the measure.

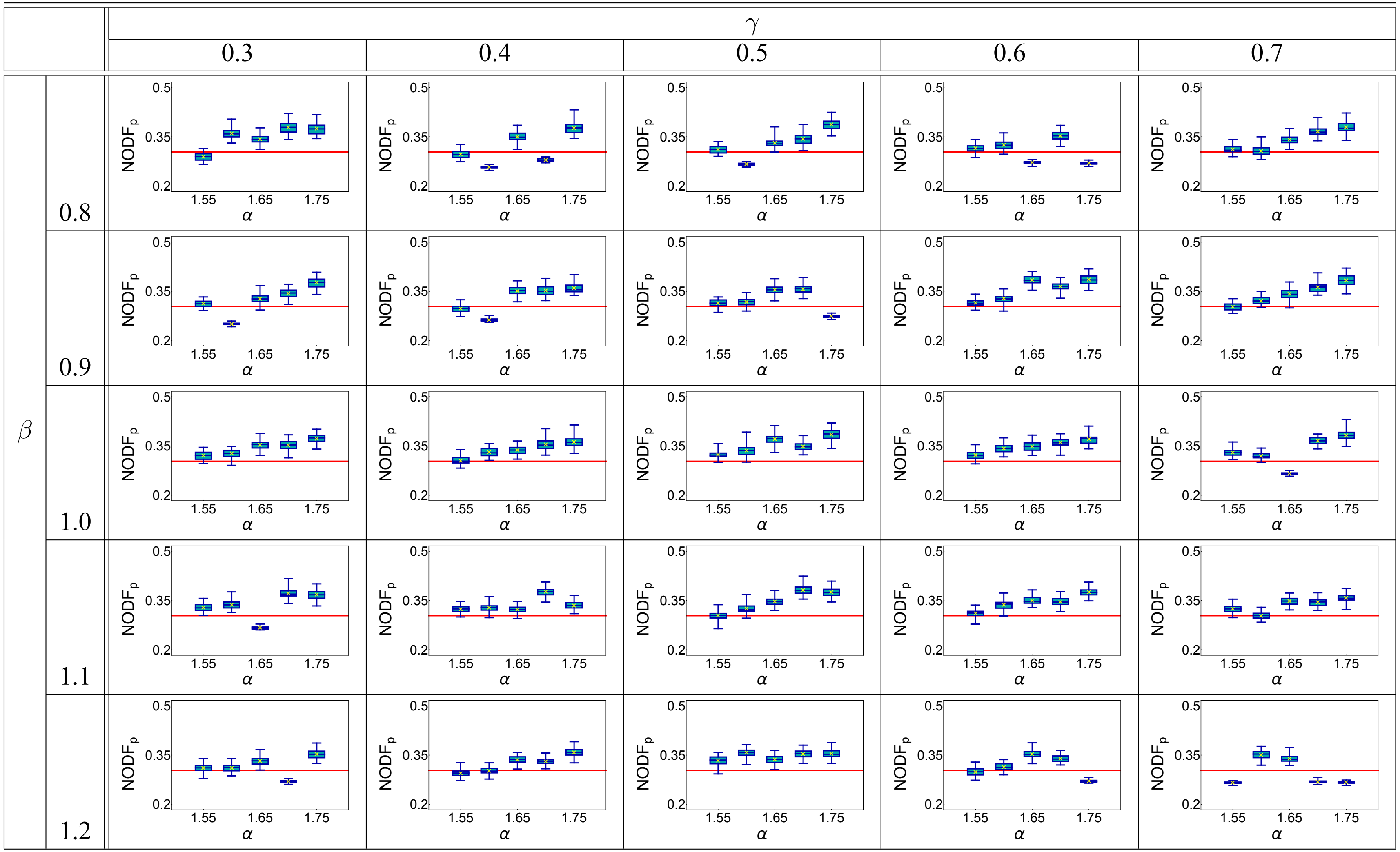

Table S2: Parameters space analysis: $\mathbf{NODF_p}$. It is possible to observe the variation of the NODF$_p$ at the changing of the parameters α, $\beta\,\gamma$; the parameter k^0 has been kept fixed to the value 4, since no variation in any of the measure analysed has been observed for greater values. The NODF$_p$ measured on the original matrix is represented as a red line. The best values of the parameter α for reproducing NODF$_p$ are the lowest analysed, i.e. $\alpha \leq 1.65$. Instead, the wider area of acceptance for the γ parameter is for the central area of the table, i.e. $0.4 \leq \gamma \leq 0.6$, while β is more or less non-influential on the the acceptance of the measure. Note that all the graphs presented here are completely overlapping with the one of the Table S1, because for the analysed network the approximation of Eq.(6) holds.

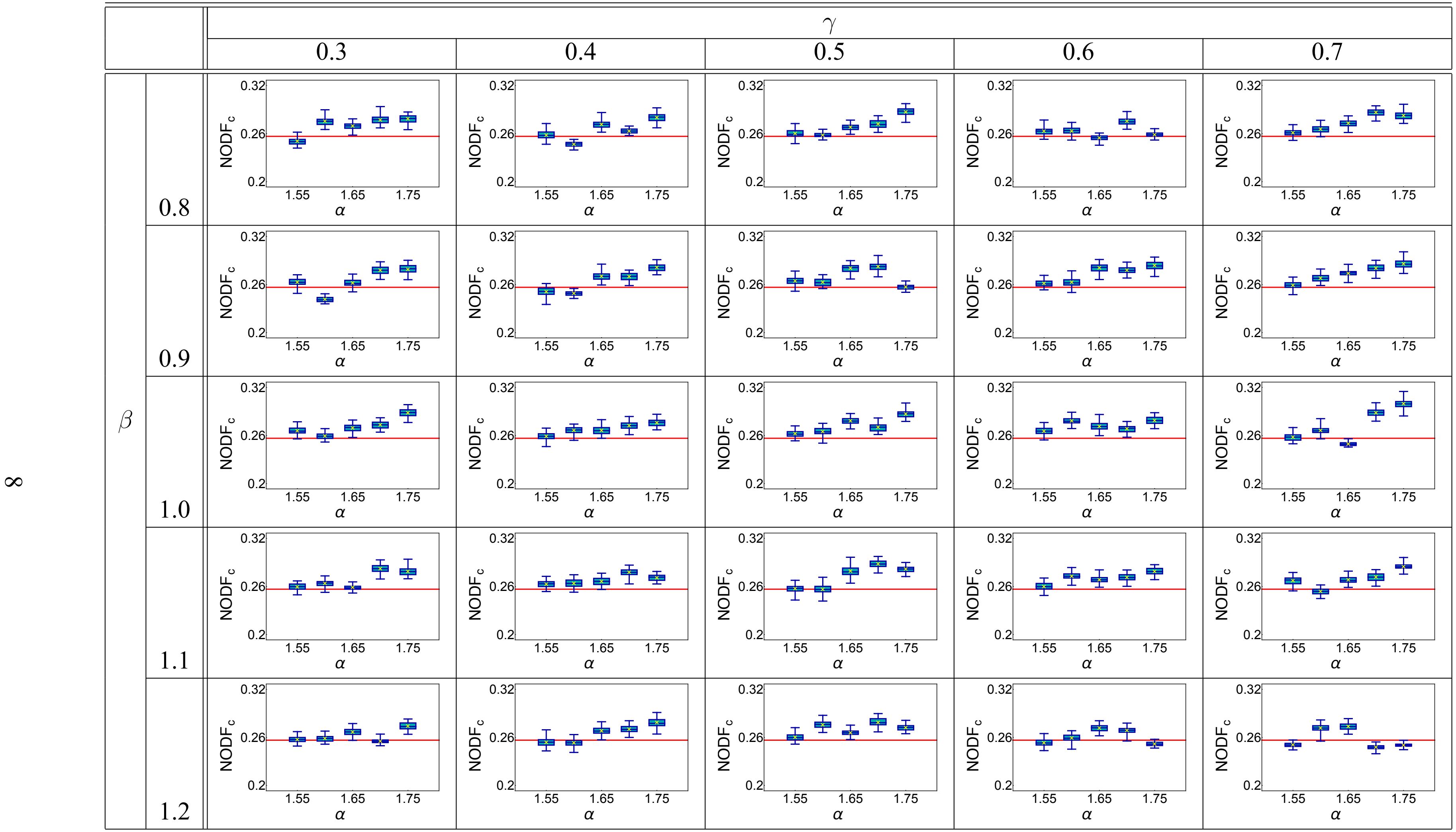

Table S3: Parameters space analysis: **$\mathbf{NODF_c}$**. It is possible to observe the variation of the NODF$_c$ at the changing of the parameters α, β γ; the parameter k^0 has been kept fixed to the value 4, since no variation in any of the measure analysed has been observed for greater values. The NODF$_c$ measured on the original matrix is represented as a red line. The best values of the parameter α are the lowest analysed, i.e. $\alpha \leq 1.65$, as in Table S2. In replicating the measure of NODF$_c$ the value of β and γ are more non influential than in Table S2 and more or less all configuration (α, β, γ) are able to correctly replicate the real value.

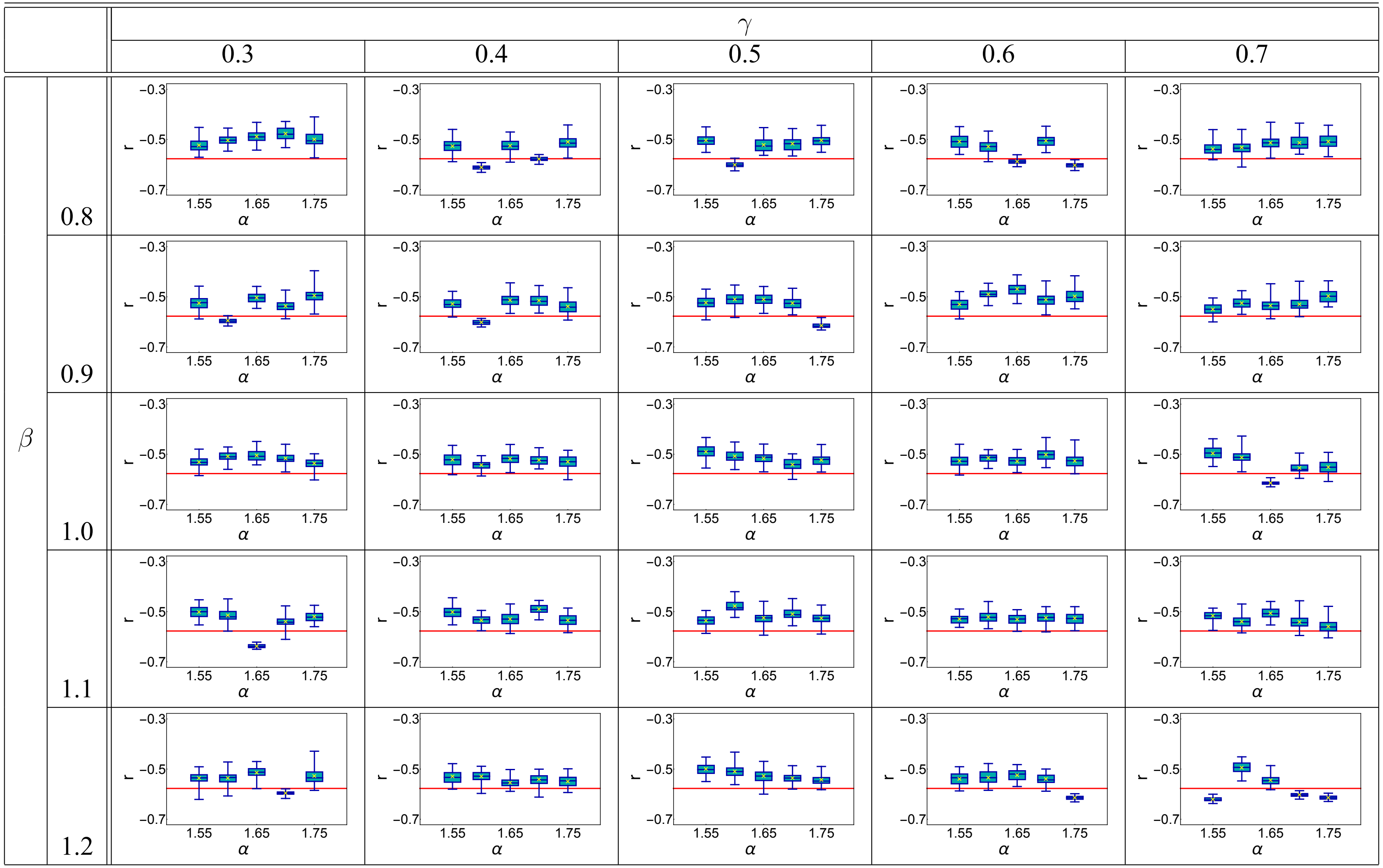

Table S4: Parameters space analysis: It is possible to observe the variation of the r at the changing of the parameters α, $\beta\,\gamma$; the parameter k^0 has been kept fixed to the value 4, since no variation in any of the measure analysed has been observed for greater values. The r measured on the original matrix is represented as a red line. Instead, the wider area of acceptance for the β and γ parameters is around the "anti-diagonal" of the table represent, so high value of γ for low β and vice versa, while α is always centred over 1.65.

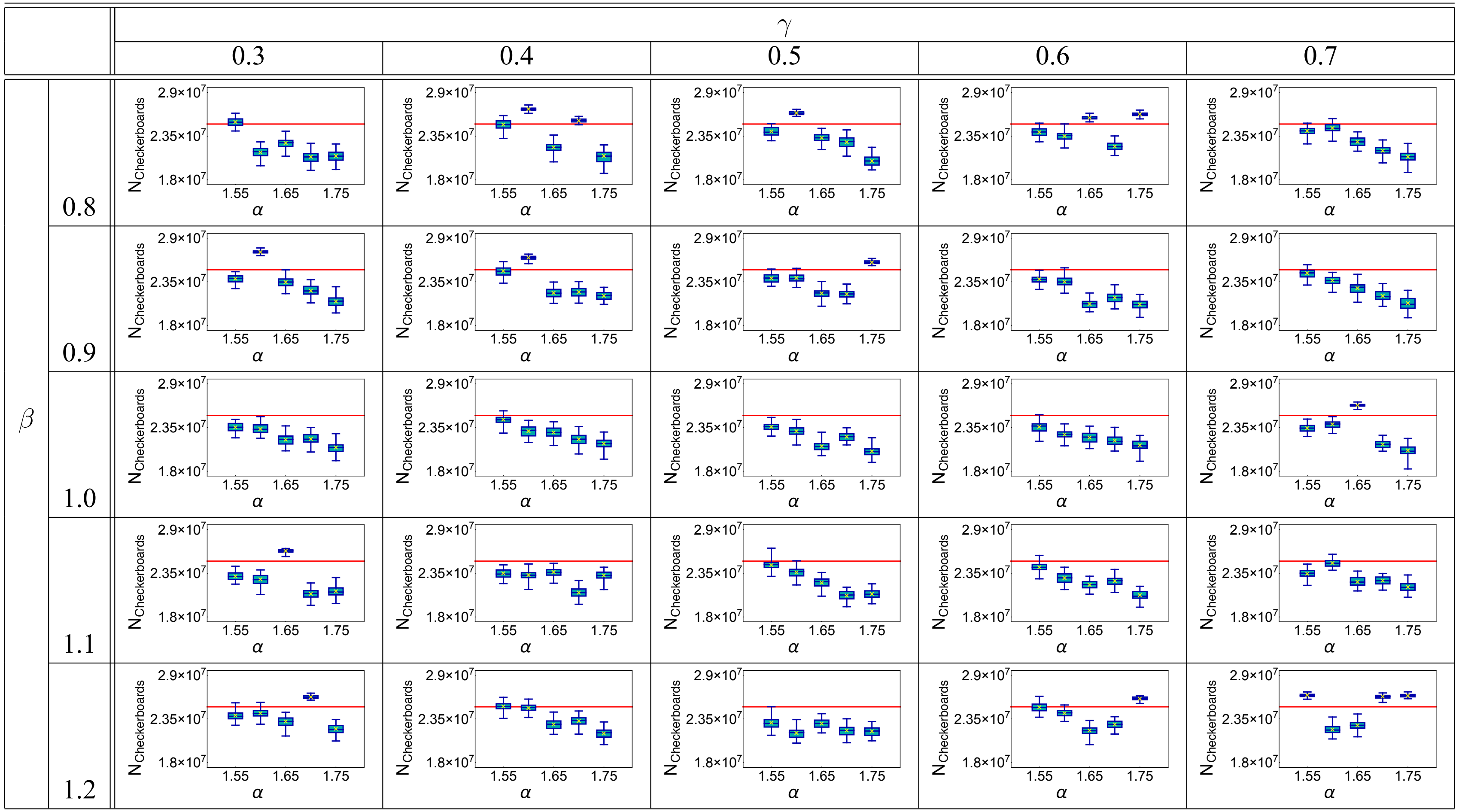

Table S5: Parameters space analysis: $\mathbf{N_{Checkerboards}}$. It is possible to observe the variation of the $N_{\text{Checkerboards}}$ at the changing of the parameters α, β γ; the parameter k^0 has been kept fixed to the value 4, since no variation in any of the measure analysed has been observed for greater values. The $N_{\text{Checkerboards}}$ measured on the original matrix is represented as a red line. The wider area of acceptance for the β and γ parameters is similar to the one of the Table S4, while αs performing better are a little bit lower, say $\alpha \leq 1.65$.

		γ				
		0.3	0.4	0.5	0.6	0.7
β	0.8	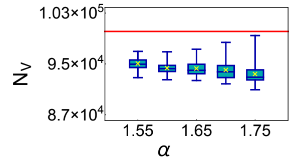	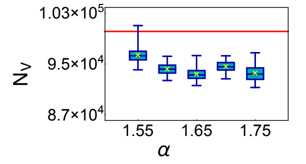	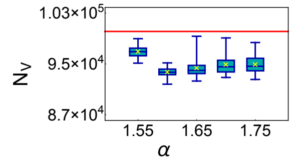	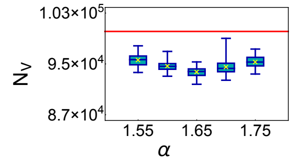	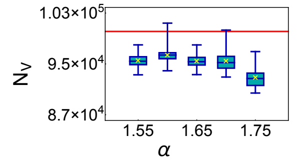
	0.9	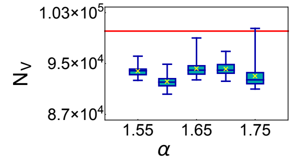	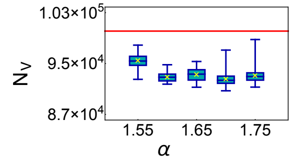	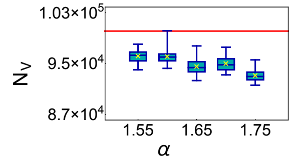	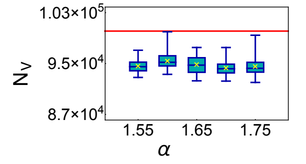	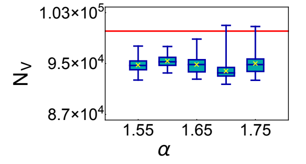
	1.0	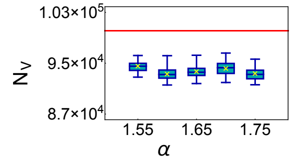	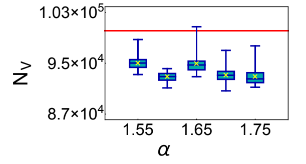	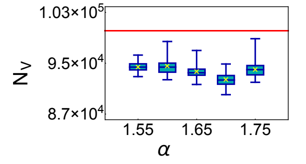	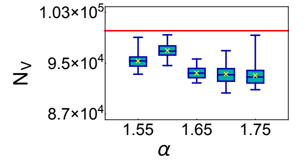	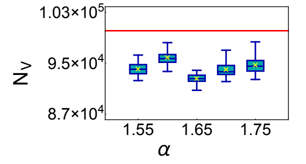
	1.1	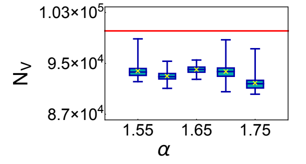	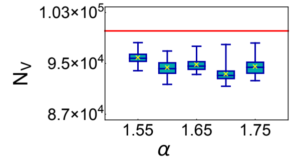	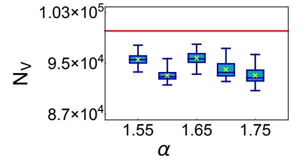	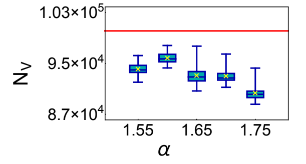	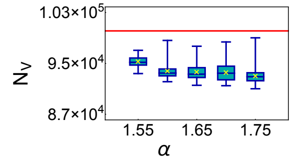
	1.2	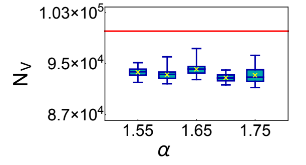	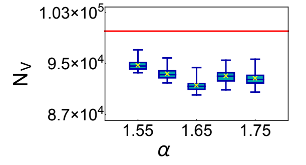	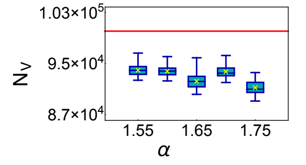	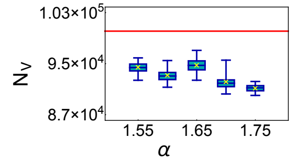	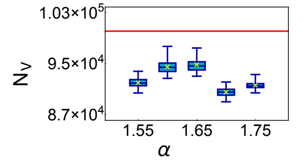

Table S6: Parameters space analysis: $\mathbf{N_V}$. It is possible to observe the variation of the N_V at the changing of the parameters $\alpha,\ \beta\,\gamma$; the parameter k^0 has been kept fixed to the value 4, since no variation in any of the measure analysed has been observed for greater values. As it is possible to see in the present table, our model is not able to capture the number of $V-$motifs in the network for more or less none of the parameters analysed. This phenomenon is due to the fact that the model evolution is based on a hierarchical structure for products (the products network) that is not present for the countries: in effect, as Table S7 shows, there is much more agreement with the original data for the $\Lambda-$motifs.

		γ				
		0.3	0.4	0.5	0.6	0.7
β	0.8	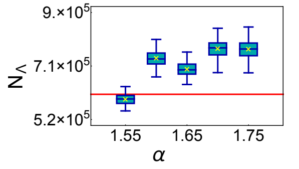	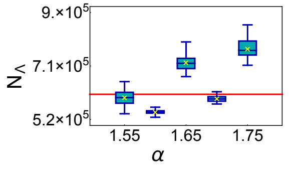	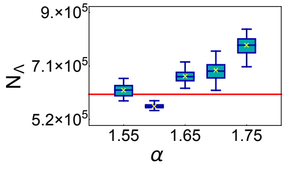	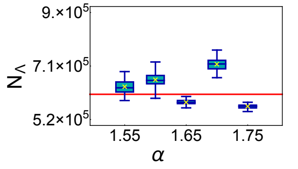	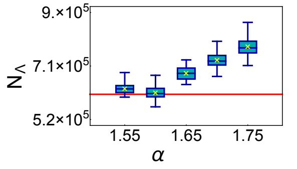
	0.9	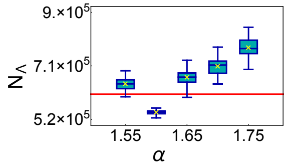	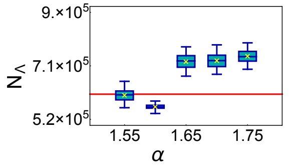	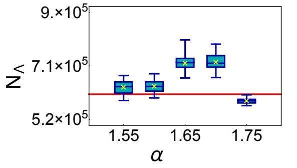	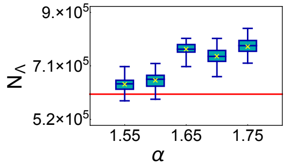	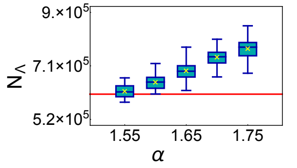
	1.0	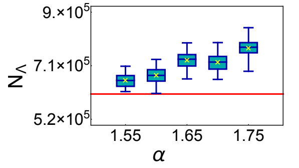	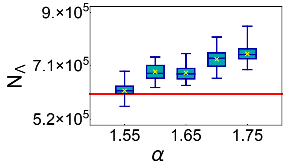	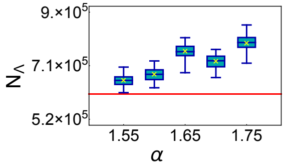	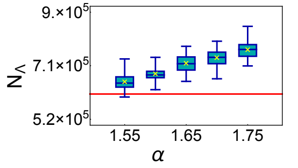	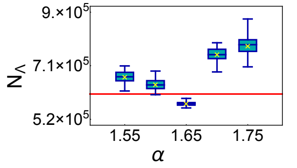
	1.1	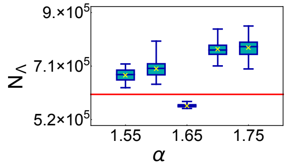	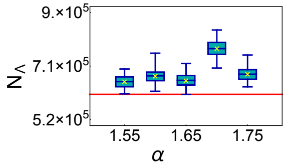	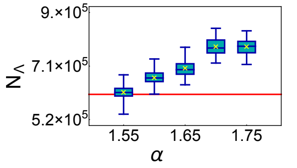	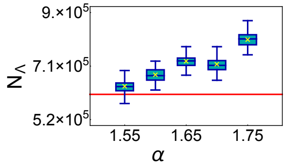	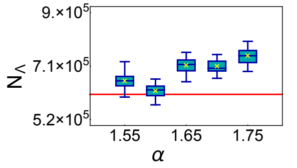
	1.2	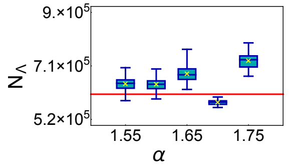	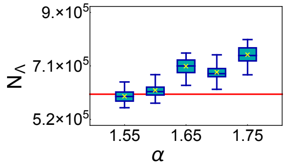	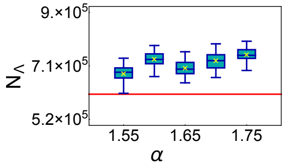	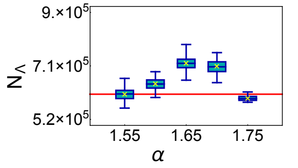	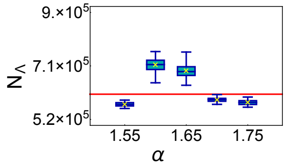

Table S7: Parameters space analysis: $\mathbf{N}_{\Box}$. It is possible to observe the variation of the N_{Λ} at the changing of the parameters α, β γ; the parameter k^0 has been kept fixed to the value 4, since no variation in any of the measure analysed has been observed for greater values. Respect to the Table S6 we find a better agreement in reproducing $\Lambda-$motifs: this fact is probably due to base the evolution of the model on a (evolving) structure for products, which keeps trace in the total number of $\Lambda-$motif, i.e. the number of co-occurrence of 2 different products in the exports baskets. As in the previous tables, the best results are obtained for low value of α and the area along the "anti-diagonal" of the presented table.

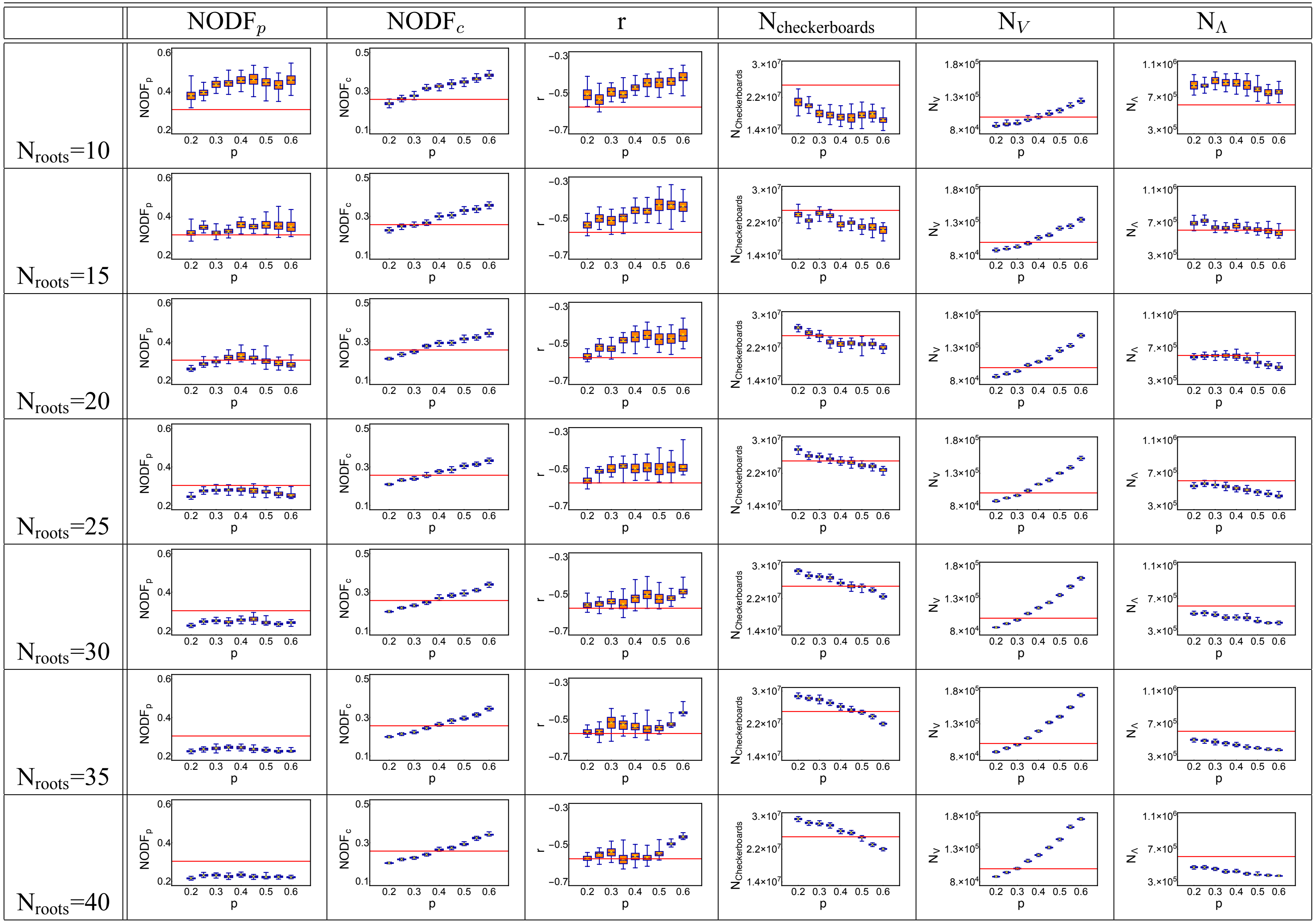

Table S8: Initial Conditions analysis: in the Table S8 it is possible to observe how the measurements vary in changing the initial condition (for fixed parameter: $\alpha = 1.6$, $\beta = 1$, $\gamma = 0.6$, $k^0 = 4$) as compared with the values from the real matrix (the red line). Every boxplot contains the distribution of 56 simulations. Initial conditions are performed assigning N_{roots} initial products roots, each with probability P_0. As it is possible to see, the best agreement for most of the measures used is for $10 \leq N_{\text{roots}} \leq 25$ and $P_0 \leq 0.4$.

References

[1] Caldarelli G, Cristelli M, Gabrielli A, Pietronero L, Scala A, Tacchella A. A Network Analysis of Countries' Export Flows: Firm Grounds for the Building Blocks of the Economy. PLoS ONE. 2012 Oct;7:47278.

[2] Tacchella A, Cristelli M, Caldarelli G, Gabrielli A, Pietronero L. A new metrics for countries' fitness and products' complexity. Scientific reports. 2012;2.

[3] Tacchella A, Cristelli M, Caldarelli G, Gabrielli A, Pietronero L. Economic complexity: conceptual grounding of a new metrics for global competitiveness. Journal of Economic Dynamics and Control. 2013;37(8):1683--1691.

[4] Cristelli M, Gabrielli A, Tacchella A, Caldarelli G, Pietronero L. Measuring the Intangibles: A Metrics for the Economic Complexity of Countries and Products. PloS one. 2013;8(8):e70726.

[5] Hidalgo CA, Klinger B, Barabási AL, Hausmann R. The product space conditions the development of nations. Science. 2007;317(5837):482--487.

[6] Hidalgo CA, Hausmann R. The building blocks of economic complexity. Proceedings of the National Academy of Sciences. 2009;106(26):10570--10575.

[7] Pugliese E, Zaccaria A, Pietronero L. On the convergence of the Fitness-Complexity Algorithm. ArXiv e-prints. 2014 Oct;.

[8] Almeida-Neto M, Guimaraes P, Jr PRG, Loyola RD, Ulrich W. A consistent metric for nestedness analysis in ecological systems : Reconciling concept and measurement. Oikos. 2008;117(8):1227--1239.

[9] Bastolla U, Fortuna MA, Pascual-Garcia A, Ferrera A, Luque B, Bascompte J. The architecture of mutualistic networks minimizes competition and increases biodiversity. Nature. 2009;458(7241):1018--1020.

[10] Suweis S, Simini F, Banavar JR, Maritan A. Emergence of structural and dynamical properties of ecological mutualistic networks. Nature. 2013 Aug;500(7463):449--452.

[11] Johnson S, Dominguez-Garcia V, Munoz MA. Factors determining nestedness in complex networks. ArXiv e-prints. 2013 Jul;.

[12] Zaccaria A, Cristelli M, Tacchella A, Pietronero L. How the Taxonomy of Products Drives the Economic Development of Countries. PloS one. 2014;9(12):e113770.

[13] Hausmann R, Klinger B. The structure of the product space and the evolution of comparative advantage. Center for International Development at Harvard University; 2007.

[14] Staniczenko PP, Kopp JC, Allesina S. The ghost of nestedness in ecological networks. Nature communications. 2013;4:1391.

[15] Newman M. Assortative Mixing in Networks. Physical Review Letters. 2002;89(20):208701.

[16] Stone L, Roberts A. The checkerboard score and species distributions. Oecologia. 1990;85(1):74--79.

[17] Saracco F, Di Clemente R, Gabrielli A, Squartini T. Randomizing bipartite networks: the case of the World Trade Web. Sci Rep. 2015 06;5.

[18] Saracco F, Di Clemente R, Gabrielli A, Squartini T. Detecting the bipartite World Trade Web evolution across 2007: a motifs-based analysis. arXiv preprint arXiv:150803533. 2015;.
